# Unsupervised home use of an overnight closed‐loop system over 3–4 weeks: a pooled analysis of randomized controlled studies in adults and adolescents with type 1 diabetes

**DOI:** 10.1111/dom.12427

**Published:** 2015-01-09

**Authors:** H. Thabit, D. Elleri, L. Leelarathna, J. M. Allen, A. Lubina‐Solomon, M. Stadler, E. Walkinshaw, A. Iqbal, P. Choudhary, M. E. Wilinska, K. D. Barnard, S. R. Heller, S. A. Amiel, M. L. Evans, D. B. Dunger, R. Hovorka

**Affiliations:** ^1^ Wellcome Trust‐MRC Institute of Metabolic ScienceUniversity of CambridgeCambridgeUK; ^2^ Academic Unit of Diabetes, Endocrinology and Metabolism, Department of Human MetabolismUniversity of SheffieldSheffieldUK; ^3^ Diabetes Research GroupKings College LondonLondonUK; ^4^ Human Development and Health Academic UnitFaculty of Medicine, University of SouthamptonSouthamptonUK

**Keywords:** clinical trial, glycaemic control, closed‐loop insulin delivery, type 1 diabetes

## Abstract

**Aims:**

To compare overnight closed‐loop and sensor‐augmented pump therapy in patients with type 1 diabetes by combining data collected during free‐living unsupervised randomized crossover home studies.

**Methods:**

A total of 40 participants with type 1 diabetes, of whom 24 were adults [mean ± standard deviation (s.d.) age 43 ± 12 years and glycated haemoglobin (HbA1c) 8.0 ± 0.9%] and 16 were adolescents (mean ± s.d. age 15.6 ± 3.6 years and HbA1c 8.1 ± 0.8%), underwent two periods of sensor‐augmented pump therapy in the home setting, in combination with or without an overnight closed‐loop insulin delivery system that uses a model predictive control algorithm to direct insulin delivery. The order of the two interventions was random; each period lasted 4 weeks in adults and 3 weeks in adolescents. The primary outcome was time during which sensor glucose readings were in the target range of 3.9–8.0 mmol/l.

**Results:**

The proportion of time when sensor glucose was in the target range (3.9–8.0 mmol/l) overnight (between 24:00 and 08:00 hours) was 18.5% greater during closed‐loop insulin delivery than during sensor‐augmented therapy (p < 0.001). Closed‐loop therapy significantly reduced mean overnight glucose levels by 0.9 mmol/l (p < 0.001), with no difference in glycaemic variability, as measured by the standard deviation of sensor glucose. Time spent above the target range was reduced (p = 0.001), as was time spent in hypoglycaemia (<3.9 mmol/l; p = 0.014) during closed‐loop therapy. Lower mean overnight glucose levels during closed‐loop therapy were brought about by increased overnight insulin delivery (p < 0.001) without changes to the total daily delivery (p = 0.84).

**Conclusion:**

Overnight closed‐loop insulin therapy at home in adults and adolescents with type 1 diabetes is feasible, showing improvements in glucose control and reducing the risk of nocturnal hypoglycaemia.

## Introduction

Intensive insulin therapy reduces the risks of macro‐ and microvascular complications in type 1 diabetes 1, 2 but is limited by hypoglycaemia, a key barrier to achieving tight glycaemic control 3. Nocturnal hypoglycaemia remains a significant concern because of its morbidity and link with sudden unexpected death in type 1 diabetes 4, 5. Achieving persistent euglycaemia safely overnight may help reduce the risk and burden to patients, carers and their families.

The closed‐loop system delivers insulin in a continually glucose‐responsive manner by combining subcutaneous continuous glucose monitoring and subcutaneous insulin pump delivery 6. This novel approach differs from conventional insulin pump therapy in its use of a control algorithm, which automatically directs insulin delivery based on real‐time sensor glucose levels. Randomized controlled trials in research facility settings in adults and children with type 1 diabetes have reported improvements in overnight glucose control whilst reducing the risk of nocturnal hypoglycaemia 7, 8, 9 and transitional studies, characterized by close monitoring in diabetes camp and hotel settings, have paved the way for closed‐loop system use in out‐of‐hospital settings 10, 11.

Unsupervised home studies constitute the ultimate ‘testbed’, as investigations under free‐living conditions provide unequivocal assessment of closed‐loop performance and its usability in the target environment. To increase our understanding of the benefits of unsupervised overnight closed‐loop therapy across a wide age range, we pooled data from two recent home studies in adolescents and adults with type 1 diabetes 12, 13. We were particularly interested to ascertain if closed‐loop therapy reduced time spent in hypoglycaemia, something which the two individual studies failed to detect because of their limited sample size and low background rates of nocturnal hypoglycaemia.

## Participants and Methods

### Participants and Experimental Protocol

Data were pooled from adult and adolescence overnight closed‐loop studies performed in unsupervised settings in the patient's home 12, 13. Between 1 July 2012 and 23 December 2013, adults aged ≥18 years were enrolled from diabetes clinics at Addenbrooke's Hospital, Cambridge, Sheffield Teaching Hospitals, Sheffield and Kings College Hospital, London, and adolescents aged 12–18 years were enrolled from Paediatric Diabetes Clinics at Addenbrooke's Hospital, Cambridge and University College Hospital, London. Inclusion criteria were: type 1 diabetes (World Health Organization criteria); >1 year from diagnosis or confirmed C‐peptide‐negative; insulin pump therapy for ≥3 months; four or more capillary glucose measurements per day; and glycated haemoglobin (HbA1c) level ≤10% (86 mmol/mol). Exclusion criteria included: established nephropathy; neuropathy or proliferative retinopathy; total daily insulin dose ≥2.0 U/kg; regular use of continuous glucose monitoring <1 month before enrolment; severe visual or hearing impairment; and pregnancy or breast feeding.

After a 2–4‐week run‐in phase, participants applied insulin pump therapy with real‐time continuous glucose monitoring at home on two periods with or without overnight closed‐loop therapy. Each period lasted 4 (adults) or 3 (adolescents) weeks. Identical insulin pump and real‐time continuous glucose monitoring devices were used during the two study periods, which were separated by a 3–4‐week washout period, during which participants used their own pump and discontinued continuous glucose monitoring.

Signed informed consent was obtained from all participants aged ≥16 years, and from parents or guardians of participants aged <16 years (assent was obtained from minors). Both study protocols were approved by the East of England Central Cambridge Ethics Committee. The trials were registered under Clinical Trials numbers NCT01440140 and NCT01221467.

### Randomization and Blinding

The order of the two study periods was random and was determined after the run‐in phase using computer‐generated permuted block randomization. During the run‐in phase, the continuous glucose monitor receiver was modified to mask the recorded sensor glucose concentrations. Downloaded sensor glucose readings were used to optimize the study insulin pump therapy before randomization. Participants had access to sensor glucose readings after the end of the run‐in phase.

### Study Procedures

Blood samples for plasma glucose and C‐peptide measurements were taken after enrolment. Participants were trained on the features of the study insulin pump (Dana R Diabecare; Sooil, Seoul, South Korea) and continuous glucose monitoring (FreeStyle Navigator; Abbott Diabetes Care, Alameda, CA, USA). After the run‐in period, compliance was demonstrated by the use the continuous glucose monitor for at least 14 (adults) and 8 days (adolescents). Participants used the rapid‐acting insulin analogue normally used in their usual clinical care and the built‐in bolus wizard of the insulin pump during both interventions to calculate insulin boluses at mealtimes and when administering correction boluses. Usual basal insulin delivery settings were also applied on the study pump.

The continuous glucose monitoring device was calibrated according to the manufacturer's instructions. The sensor glucose threshold alarm for hypoglycaemia was initially set at 3.5 mmol/l and could be modified by participants. During the closed‐loop period, overnight insulin delivery was automatically directed by a closed‐loop model predictive control algorithm 14.

The adult participants spent the first closed‐loop night in the local clinical research facility and received training in the use of the closed‐loop system. In the adolescence cohort, closed‐loop training was provided to the participants and parents at home during the first closed‐loop night. Training lasted for 60–90 min and covered initiation and discontinuation of the closed‐loop system and problem troubleshooting. Participants were instructed to start the system at home after their evening meal, and to discontinue it before breakfast the next morning. Participants were trained to check capillary glucose versus sensor glucose values (calibration check) before evening meals; if sensor glucose was greater than capillary glucose by >3 mmol/l, the continuous glucose monitor was recalibrated and the calibration check was repeated before starting the closed‐loop system. These instructions reduced the risk of sensor error and the calibration check approach, expected to occur infrequently once every 3–4 weeks, was effective when assessed by computer modelling 15.

No further supervision took place over the following nights and participants used the system fully unsupervised. Participants were not restricted in dietary intake or daily activities. A 24‐h telephone support service assisted participants in clinical or technical issues that arose during the study. All participants were given troubleshooting literature and user manuals for all study devices. Standard local hypoglycaemia and hyperglycaemia treatment guidelines were followed. The data from the study insulin pumps and continuous glucose monitoring devices were downloaded by the research team at the end of study periods. In addition, during closed‐loop therapy, encrypted data from the closed‐loop system was emailed by the participants to the study team once a week.

### Closed‐Loop System

The Florence automated closed‐loop system comprised a model predictive control algorithm residing on a hand‐held computer linked by cable to the continuous glucose monitoring receiver 14. Every 12 min, the treat‐to‐target algorithm calculated a new insulin infusion rate, which was automatically set on the study pump via wireless communication.

At setup, the research team entered participants' weight and total daily insulin dose on the first night during which the closed‐loop system was used. Data for carbohydrate intake, as entered by participants into the insulin pump built‐in bolus wizard, were automatically downloaded to the hand‐held computer during the use of the closed‐loop system. Insulin delivery history, including manually instructed insulin boluses, was also automatically downloaded.

### Laboratory Assays

Baseline random C‐peptide levels were measured using a chemiluminescence immunoassay (Liaison XL; DiaSorin Deutschland GmbH, Dietzenbach, Germany; interassay coefficients of variation 5.6% at 563 pmol/l, 4.5% at 2529 pmol/l and 5.8% at 5449 pmol/l). HbA1c was measured centrally with ion exchange high‐performance liquid chromatography (G8 HPLC Analyzer; Tosoh Bioscience, South San Francisco, CA, USA; interassay coefficients of variation 1.3% at 31.2 mmol/mol and 0.8% at 80.5 mmol/mol).

### Outcomes

The primary efficacy outcome was the time spent in the target glucose range (3.9–8.0 mmol/l) between 24:00 and 08:00 hours, as recorded by continuous glucose monitoring. Secondary outcomes included mean glucose concentration, time spent at glucose levels <3.9 mmol/l (hypoglycaemia) and >8.0 mmol/l (hyperglycaemia), and insulin delivery. Overnight glucose variability was assessed by the standard deviation and the coefficient of variation of continuous glucose monitoring levels. We assessed hypoglycaemia burden by calculating the glucose sensor area under the curve <3.5 mmol/l. We calculated secondary outcomes from 24:00 to 08:00 hours and over 24 h.

### Statistics

Analyses were carried out on an intention‐to‐treat basis. Normally distributed data were compared using the paired *t*‐test and non‐normally distributed data using the Wilcoxon signed‐rank test. Results are presented as mean [standard deviation (s.d.)] or median [interquartile range (IQR)], unless stated otherwise. p values <0.05 were taken to indicate statistical significance. Outcomes were calculated using gstat software (version 2.0, University of Cambridge) and statistical analyses were conducted using spss (version 21).

## Results

### Baseline Characteristics

A total of 24 adults and 16 adolescents completed both study periods. Baseline characteristics are shown in Table 1.

**Table 1:** Baseline characteristics

	Adults (n = 24)*	Adolescents (n = 16)*
Age, years	43 ± 12	15.6 ± 2.1
Gender: male/female	13/11	10/6
Body mass index, kg/m^2^	26.0 ± 3.5	22.4 ± 3.7
HbA1c		
%	8.1 ± 0.8	8.0 ± 0.9
mmol/mol	64.9 ± 8.9	63.9 ± 9.4
Duration of diabetes, years	29 ± 11	7.2 ± 4.3
Duration on pump, years	6.3 ± 4.4	3.0 ± 2.3
Total daily insulin, U/kg/day	0.5 ± 0.1	0.8 ± 0.2

HbA1c, glycated haemoglobin. Data are mean ± standard deviation unless otherwise indicated.

* All with C‐peptide levels lower than 33 pmol/l.

### Primary and Secondary Outcomes Overnight

**Figure 1:**
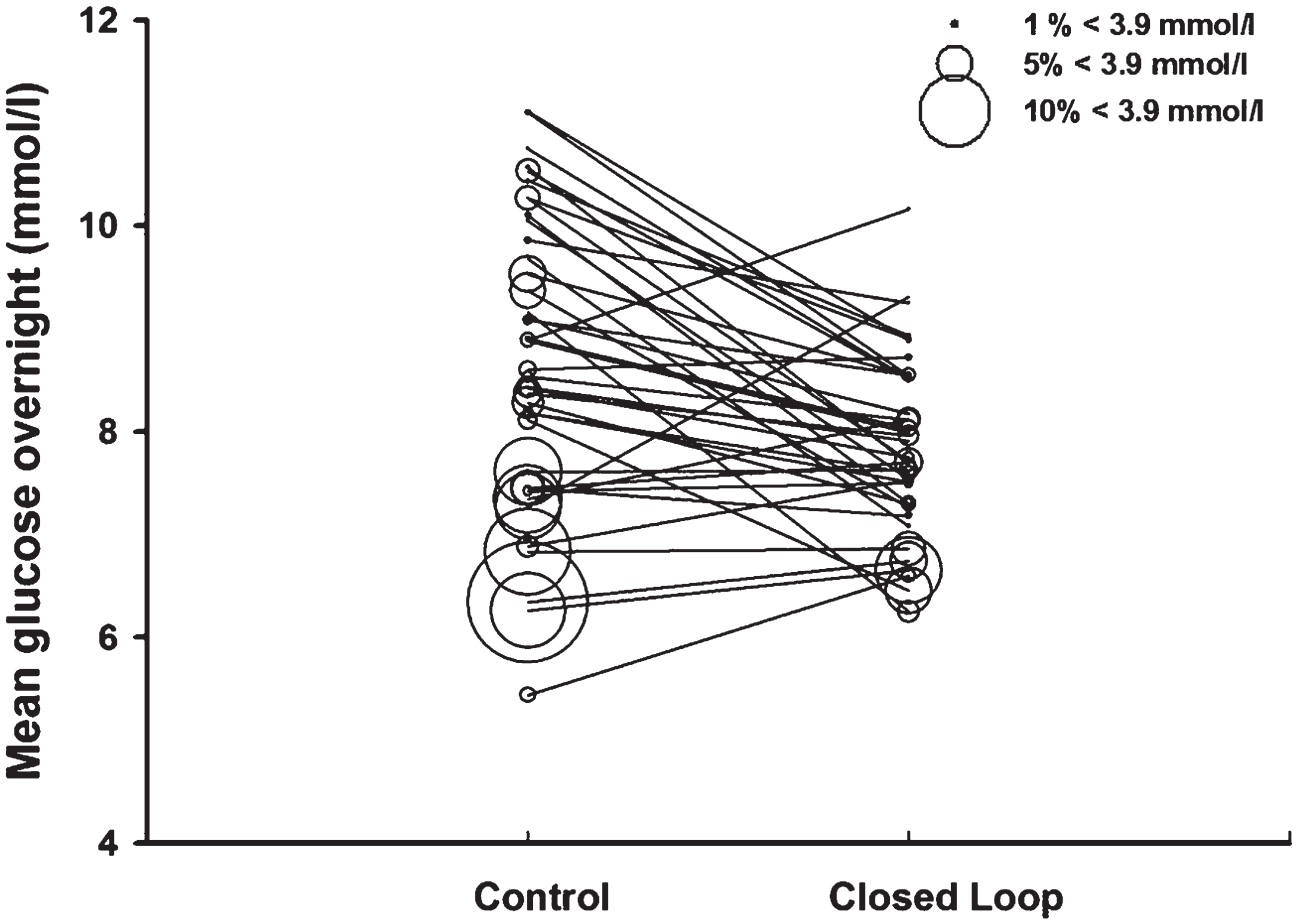
Individual values of mean glucose levels and proportion of time spent at low glucose levels (<3.9 mmol/l) in the whole cohort. Line plots show individual values of mean overnight glucose during the intention‐to‐treat analysis. The diameter of each circle is proportional to the percentage of time that each participant spent with a low glucose value <3.9 mmol/l.

The primary endpoint, the proportion of time when overnight sensor glucose levels were in the target range, was significantly increased by a mean of 18.4% [95% confidence interval (CI) 13.5–23.4%; p < 0.001] during closed‐loop therapy (Table 2). Closed‐loop therapy significantly reduced mean overnight sensor glucose by 0.9 mmol/l (95% CI 0.4–1.3 mmol/l; p < 0.001), as well as the proportion of time when sensor glucose values were in hyperglycaemia (>8.0 mmol/l; mean 15.9%, 95% CI 10.7–21.0%; p < 0.001) and hypoglycaemia (<3.9 mmol/l; median 0.9%, 95% CI 0.2–2.2; p = 0.014; Table 2, Figure 1). There was no significant difference in the burden of hypoglycaemia as measured by the area under the curve when sensor glucose levels were <3.5 mmol/l (p = 0.15).

**Table 2:** Intention‐to‐treat comparison of glucose outcomes from 24:00 to 08:00 hours during overnight closed‐loop therapy and the control period in the home setting over 3–4 weeks

	**Closed‐loop period (n = 40)**	**Control period (n = 40)**	**p**
Mean ± s.d. glucose, mmol/l	7.9 ± 0.9	8.7 ± 1.4	<0.001
Mean ± s.d. within‐night standard deviation of glucose, mmol/l	2.0 ± 0.3	1.9 ± 0.3	0.47
Mean ± s.d. within‐night coefficient of variation of glucose, %	25.2 ± 3.5	22.8 ± 5.0	0.023
Mean ± s.d. between‐nights coefficient of variation of glucose, %	24.8 ± 6.9	29.1 ± 6.9	0.002
Mean ± s.d. proportion of time spent at glucose level, %			
3.9–8.0 mmol/l*	59.2 ± 11.5	40.7 ± 13.4	<0.001
3.9–10.0 mmol/l	77.4 ± 8.6	61.8 ± 13.3	<0.001
>8.0 mmol/l	37.9 ± 12.4	53.8 ± 17.0	0.001
Median (IQR) proportion of time spent at glucose level, %			
>16.7 mmol/l	0.66 (0.0, 2.3)	1.3 (0.0, 2.8)	0.15
<3.9 mmol/l	1.9 (0.7, 3.5)	2.9 (1.0, 6.4)	0.014
<3.5 mmol/l	0.7 (0.3, 2.0)	1.1 (0.21, 3.9)	0.098
<2.8 mmol/l	0.2 (0.0, 0.6)	0.2 (0.0, 1.3)	0.29
Median (IQR) AUC_day_ <3.5 mmol/l, mmol/l × min	4.7 (1.2, 16.9)	6.4 (0.4, 33.8)	0.15
Mean ± s.d. glucose at 24:00 hours, mmol/l	9.1 ± 1.3	9.0 ± 1.8	0.39
Mean ± s.d. glucose at 08:00 hours, mmol/l	7.7 ± 0.92	9.0 ± 1.4	<0.001

AUC_day_, Glucose area under curve below 3.5 mmol/l per day; IQR, interquartile range; s.d., standard deviation.

* Primary endpoint.

Glucose variability overnight measured as standard deviation was similar during the two interventions. The mean ± s.d. coefficient of variation of overnight glucose within each night was higher during closed‐loop than during sensor‐augmented pump therapy (25.2 ± 3.5% vs. 22.8 ± 5.0%; p = 0.023; Table 2). Conversely, the between‐nights coefficient of variation of overnight glucose was significantly reduced by closed‐loop therapy (24.8 ± 6.9% vs. 29.1 ± 6.9%; p = 0.002).

Exploratory per‐protocol analysis, considering nights when the closed‐loop system was turned on for at least 6 h (closed‐loop period) or sensor data were available for at least 6 h (control period), showed that closed‐loop therapy increased the proportion of time overnight spent in target by 20.7% (95% CI 15.7–25.6%; p < 0.001; Table 3). This was achieved by reducing mean glucose levels by 1.0 mmol/l (95% CI 0.5–1.4 mmol/l; p < 0.001) as well as reducing time spent below target overnight by a median of 3.2% (95% CI 0.8–5.0%; p = 0.005).

**Table 3:** Comparison of glucose outcomes from 24:00 to 08:00 hours, with at least 6 h of closed‐loop operation (intervention group) and 6 h of sensor data (control group)

	Closed‐loop group (n = 40)	Control group (n = 40)	p
Number of nights	786	881	
Mean ± s.d. glucose, mmol/l	7.8 ± 0.96	8.7 ± 1.4	<0.001
Mean ± s.d. proportion of time spent at glucose level 3.9–8.0 mmol/l, %	61.3 ± 12.5	40.6 ± 13.4	<0.001
Median (IQR) proportion of time spent at glucose level <3.9 mmol/l, %	1.1 (0.58, 2.9)	2.9 (0.8, 5.8)	0.005
Median (IQR) AUC_day_ <3.5 mmol/l, mmol/l × min	2.5 (0.34, 10.1)	6.2 (0.21, 33.8)	0.09
Mean ± s.d. lucose at 24:00 hours, mmol/l	9.0 ± 1.3	9.0 ± 1.9	0.69
Mean ± s.d. lucose at 08:00 hours, mmol/l	7.7 ± 1.1	9.0 ± 1.3	<0.001

AUC_day_, Glucose area under curve below 3.5 mmol/l per day; IQR, interquartile range; s.d., standard deviation.

### Secondary Outcomes From Midnight to Midnight

Endpoints calculated over 24 h showed that the overnight closed‐loop system significantly increased the proportion of time spent within a wider target range of 3.9–10.0 mmol/l by a mean of 4.7% (95% CI 2.0–7.5%; p = 0.001; Table 4). Overnight closed‐loop therapy reduced the overall mean sensor glucose level by a mean of 0.5 mmol/l (95% CI 0.1–1.0 mmol/l; p = 0.016). This was achieved by significantly reducing both proportion of time spent over 24 h with glucose above the target by a mean of 7.0% (95% CI 3.6–10.3%; p < 0.001) and by reducing the proportion of time spent in hypoglycaemia by a median of 1.6% (95% CI 0.4–2.8%; p = 0.015).

**Table 4:** Comparison of 24‐h glucose outcomes during overnight closed‐loop therapy and the control period in the home setting over 3–4 weeks

	Closed‐loop period (n = 40)	Control period (n = 40)	p
Mean ± s.d. glucose, mmol/l	8.6 ± 1.2	9.1 ± 1.4	0.016
Mean ± s.d. proportion of time spent at glucose level, %			
3.9–10.0 mmol/l	63.6 ± 9.9	58.8 ± 10.5	0.001
>10.0 mmol/l	29.5 ± 10.5	36.5 ± 13.3	<0.001
Median (IQR) proportion of time spent at glucose level <3.9 mmol/l, %	1.8 (0.91, 3.2)	2.7 (1.3, 5.7)	0.015
Median (IQR) AUC_day_ < 3.5 mmol/l, mmol/l × min	5.2 (2.0, 12.1)	6.9 (2.4, 29.5)	0.078

AUC_day_, Glucose area under curve below 3.5 mmol/l per day; IQR, interquartile range; s.d., standard deviation.

### Insulin Requirements and Utility of the Closed‐Loop System

The closed‐loop system delivered 17% more insulin overnight (p < 0.001; Table 5). The total daily insulin delivered was similar during the two interventions (p = 0.84). Adult and adolescent participants used closed‐loop therapy of their own volition for 87% (856 nights) of the whole study duration (Table S1). Closed‐loop therapy was used for a median of 9 h at home each night; the median closed‐loop therapy start time was 22:37 hours and the stop time was 07:30 hours. The causes and frequency of unintentional closed‐loop interruptions are listed in Table S1.

**Table 5:** Insulin delivery overnight (24:00–08:00 hours) and over 24 h

	Closed‐loop period, n = 40	Control period, n = 40	p
	Median (IQR)	Median (IQR)	
Overnight insulin delivery, U	7.0 (5.4, 9.3)	6.0 (4.7, 7.4)	<0.001
Total daily insulin delivery, U	40.3 (32.9, 52.6)	39.4 (32.8, 55.8)	0.84
s.d. of overnight insulin delivery, U	0.6 (0.5, 0.8)	0.1 (0.07, 0.2)	<0.001

IQR, interquartile range; s.d., standard deviation.

### Adverse Events

There were two episodes of hyperglycaemia associated with ketosis (blood ketones >1.5 mmol/l) during the control period, and one occasion during the closed‐loop period. Two episodes of severe hypoglycaemia occurred during the closed‐loop period in two adults who each had a history of hypoglycaemia unawareness, and both events happened at a time when the closed‐loop system was not operational. *Post hoc* analysis showed that closed‐loop operation was interrupted 1 h before these two episodes occurred because of disrupted wireless connectivity with the insulin pump. Before this interruption, insulin delivery had already been suspended by the algorithm because of predicted low glucose concentrations. In 1 participant, the event was probably compounded by increased physical activity during the day. User error, that is, overdelivery of insulin whilst changing infusion set overnight, was deemed to be the likely contributory cause in the second participant. Both participants recovered fully with no clinical sequelae.

## Discussion

This pooled analysis adds to the observations in two previous studies that adults and adolescents with type 1 diabetes are able to use closed‐loop therapy safely and effectively overnight at home over an extended period, without the need for remote monitoring or close supervision. The combined evaluation of the two aforementioned studies 12, 13 provides greater power and allows comprehensive assessment of closed‐loop benefits across a wide age range. A clinically significant reduction in overnight glucose was observed accompanied by reduced time spent in a hypoglycaemic state. Such combined effect has not been documented with any other means of intensified conventional insulin delivery in type 1 diabetes. Glucose variability between nights was reduced by closed‐loop therapy in the study, whilst increased variability within each night is explained by similarly elevated sensor glucose levels at midnight during both interventions, accompanied by consistent lower morning glucose at 08:00 hours during closed‐loop therapy.

Glucose concentrations remained lower over 24 h, suggesting there are extended benefits of improved overnight glucose control even when closed‐loop therapy is stopped. Furthermore, fasting morning glucose levels were 14% lower after overnight closed‐loop therapy. As a result, participants were able to administer fewer insulin boluses and corrections during the day. This may explain the similar total daily insulin dosage for both study periods, in spite of the 17% increase in overnight insulin delivery during closed‐loop.

Real‐time continuous glucose monitoring is associated with a mean HbA1c reduction of 0.5% (5.5 mmol/mol) 16, 17, 18 and an unchanged risk of nocturnal hypoglycaemia 19. Adolescents appear to benefit less, owing to a reduction in time when the glucose sensor was used 20. Frequent insulin dosing and infusion rate adjustments to match glycaemia levels and day‐to‐day variations in insulin sensitivity represent significant challenges and burden to subjects, their families and healthcare professionals. A more recent technological approach, the threshold‐suspend insulin pump, interrupts insulin delivery for up to 2 h when at a low glucose threshold. Its application reduces nocturnal frequency and duration of hypoglycaemia 21, 22, but is unable to step up insulin delivery during episodes of elevated glucose levels and does not alter overall glucose control 22.

Our closed‐loop algorithm accounts for variations in insulin requirements, finely modulating insulin delivery based on real‐time sensor glucose levels and maintaining more consistent glucose levels overnight. Other closed‐loop systems have been studied in outpatient and home settings, albeit for a shorter duration or under remote monitoring and supervision 11, 23, 24, 25. Compared with standard pump therapy, subjects on a bihormonal closed‐loop system in an outpatient setting for 5 days had lower mean glucose levels and less frequent hypoglycaemic episodes 26. Overnight closed‐loop therapy in children and adolescents at a diabetes camp over 5–6 days showed significant reductions in times spent at various hypoglycaemia glucose levels, but no significant difference in the intention‐to‐treat analysis was observed for median percentage of time in the glucose range of 3.9–8.3 mmol/l 26. A single night closed‐loop intervention at a diabetes camp showed a reduction in the frequency and duration of hypoglycaemia <3.5 and 3.3 mmol/l, with a reduction of overnight median glucose by 0.8 mmol/l 11. The same group reported significant reductions in time spent with sensor glucose levels <3.9 mmol/l and an increased percentage of time spent in the target range (3.9–7.8 mmol/l) during a 6‐week single‐centre overnight closed‐loop home study 27. In contrast to the present multicentre study, however, a remote monitoring system was used and thus the research team was immediately alerted of technical failures as well as hypo‐ and hyperglycaemia events during overnight closed‐loop therapy.

The strength of the present study is the rigour of the intention‐to‐treat analysis and duration of closed‐loop use. Its distinguishing feature is free‐living unsupervised use in home settings. Other studies performed in out‐of‐hospital settings were either shorter or were performed under remote monitoring, whereby investigators could intervene clinically during episodes of significant hypo‐ or hyperglycaemia, and could manage technical faults during the study on behalf of participants. Participants in the present study were not restricted in their activities or dietary habits, and used closed‐loop therapy at home of their own volition; thus, experimentally, these studies are the first to report on the utility and efficacy of closed‐loop therapy under real‐life day‐to‐day conditions without participants being directly monitored or supervised. The randomized crossover design of the present study allowed the participants to act as their own control. We acknowledge that the findings were limited by usability of the system. Closed‐loop therapy interruptions occurred on average once every five nights. More than 60% of interruptions were attributable to disruptions to the pump wireless connectivity or loss of sensor glucose availability. Participants' positive and negative experiences and quality of life may thus have been affected by various aspects of system utility 28. The experience and utilisation rate of 87% may be further enhanced by improvements in device connectivity.

In conclusion, unsupervised use of overnight closed‐loop therapy in adults and adolescents with type 1 diabetes is feasible. Benefits include improved overnight glucose control with reduction in time spent in a hypoglycaemic state. Longer‐term studies are warranted using devices with more reliable connectivity.

## Conflict of Interest

R. H. has received speaker honoraria from Minimed Medtronic, Lifescan, Eli Lilly, BBrau, and Novo Nordisk, serves on an advisory panel for Animas, Minimed Medtronic and Eli Lilly, has received license fees from BBraun and Beckton Dickinson, and has served as a consultant to Beckton Dickinson, BBraun, Sanofi‐Aventis and Profil. S. R. H. has undertaken consultancy work for Novo Nordisk and Eli Lilly, for which his institution has received payment. He has spoken at meetings for which he has received payment from NovoNordisk, Eli Lilly and Beckton Dickinson. Medtronic has provided research support for some of his work. M. L. E. has received speaker honoraria from Eli Lilly, Animas and Abbott Diabetes Care and served on advisory panels for Medtronic, Roche, Sanofi‐Aventis and Cellnovo. P. C. declares speaker honoraria and travel support from Medtronic, Roche and Lifescan, and has undertaken consultancy for Novo Nordisk and Eli Lilly, for which his institution has received payment. He has spoken at meetings for which he has received payment from NovoNordisk, Eli Lilly and Beckton Dickinson. Medtronic has provided research support for some of his work. MEW reports receiving licensing fees from Beckton Dickinson. R. H., D. B. D. and M. E. W. report patent applications. H. T., D. E., L. L., J. M. A., A. L. S., M. S., E. W., J. M. A., A. I., K. D. B. and S. A. A. have no competing financial interests.

R. H. and H. T. had full access to all of the data in the study and take responsibility for the integrity of the data and the accuracy of the data analysis. R. H. coordinated the study. R. H., M. L. E., S. R. H., S. A. A., D. B. D., H. T., D. E., E. W., M. E. W. and K. D. B. co‐designed the studies. H. T., D. E., J. A., A. L.‐S., M. S., E. W., A. I. and P. C. were responsible for screening and enrolment of participants. H. T., D. E., L. L., J. A., A. L.‐S., M. S., E. W., A. I. and P. C. provided patient care and/or took samples and arranged informed consent from the participants. M. W. generated the random allocation sequence using a computer‐generated random code. H. T., L. L., and D. E. carried out or supported data analysis, including the statistical analyses. R. H. designed and implemented the glucose controller. H. T. drafted the manuscript. H. T. and R. H. edited the manuscript. All authors reviewed the manuscript and approved the final version.

## Supporting information


**Table S1.** Utility and failure analysis of closed‐loop operation.
