# Supplementary material for: Unsupervised home use of an overnight closed‐loop system over 3–4 weeks: a pooled analysis of randomized controlled studies in adults and adolescents with type 1 diabetes
Source: Diabetes Obes Metab. 2015 Jan 9;17(5):452–8. doi: 10.1111/dom.12427 (PMC4510702; doi:10.1111/dom.12427)
Supplement: Supplementary file 1 — Table S1. Utility and failure analysis of closed‐loop operation. [file dom0017-0452-sd1.doc]

**Table S1**. Utility and failure analysis of closed-loop operation.

| Number of nights when closed-loop turned on | **856 (87%)** |
| --- | --- |
| Time of day when closed-loop turned on | **22:37 (21:34, 23:31)*** |
| Time of day when closed-loop turned off | **07:30 (06:53, 08:36)*** |
| Duration of closed-loop operation (hours) | **9.0 (7.8, 10.2)*** |
| Total duration of closed-loop operation (h) | **7619** |
| Number of events when closed-loop interrupted (% of total interruptions) |  |
| lack of pump connectivity | **91 (51%)** |
| unable to start closed-loop cycle within 30 mins | **21 (20%)** |
| sensor data unavailability | **24 (13%)** |
| temporary infusion changed by user | **9 (5%)** |
| extended bolus changed by user | **7 (4%)** |
| handheld computer operating system failure | **7 (4%)** |
| low battery level of handheld computer | **4 (2%)** |
| handheld computer software system error | **1 (1%)** |

* Median (interquartile range) from all study nights when closed-loop turned on
